# Associations between different types of screen-based sedentary behavior and sleep among Chinese children and adolescents

**DOI:** 10.3389/fpubh.2025.1644890

**Published:** 2026-01-12

**Authors:** Wenle Chen, Qiang Xue, Junkai Zhang, Qi Wang, Mingming Guo

**Affiliations:** 1 College of Physical Education and Sport, Beijing Normal University, Beijing, China; 2 College of Education for the Future, Beijing Normal University, Zhuhai, Guangdong, China

**Keywords:** screen-based sedentary behavior, sleep, children and adolescents, Chinese, subgroup analysis

## Abstract

**Background:**

Previous studies on the relationship between screen-based sedentary behaviors (SSB) and sleep among children and adolescents have yielded inconsistent findings, particularly concerning novel screen use. Limited research has explored whether associations differ across demographic subgroups. Therefore, this study aimed to explore associations between novel SSB and sleep among Chinese children and adolescents, as well as to assess the consistency of these associations across genders, educational levels, and urban-rural residence.

**Methods:**

Data were drawn from the China Family Panel Studies (CFPS) 2020–2022, involving 3,309 children and adolescents aged 10–18 years, 1,526 females. Sleep was measured by a self-reported average daily sleep duration, with < 8 h defined as insufficient sleep. SSB frequencies, covering five activities including online gaming and WeChat usage, were categorized into three levels by frequency (1 = No, 2 = Occasional, 3 = Daily). Generalized linear logistic regression models were used to analyze associations between various SSB and sleep, and subgroup analyses were conducted to examine heterogeneous effects.

**Results:**

Compared with participants who never watched short videos, those who watched short videos daily showed a 36% lower likelihood of insufficient sleep (OR = 0.64, 95% CI: 0.49–0.84). Compared with participants who never used online shopping, those who used online shopping daily showed a 24% higher likelihood of insufficient sleep (OR = 1.24, 95% CI: 1.03–1.49). Compared with participants who never used online gaming, those who used online gaming daily showed a 19% lower likelihood of insufficient sleep (OR = 0.81, 95% CI: 0.67–0.99). No significant associations were found between insufficient sleep and WeChat usage or online learning.

**Conclusions:**

Short-video watching and online gaming may be protective factors against insufficient sleep among Chinese children and adolescents, particularly in specific subgroups (e.g., males, rural residents, and junior high school students), while daily online shopping might be a risk factor for sleep insufficiency, especially among elementary and junior high school students. WeChat usage and online learning showed no significant associations with sleep. Future policies and research should prioritize monitoring of rapidly evolving screen-based sedentary behaviors (SSB), while implementing targeted, subgroup-specific interventions to mitigate their health impacts.

## 1 Introduction

Sleep is a complex physiological process that involves the periodic modulation of neural activity to achieve physical restoration and cognitive integration (1). It is characterized by periodicity, staging, and rhythmicity. Individuals with insufficient sleep commonly exhibit impaired attention, which is frequently accompanied by academic underperformance and reduced productivity (1). Children and adolescents, as the population group with the highest sleep requirements (2), require 8–12 h of daily sleep to support neurodevelopment and emotional regulation. Insufficient sleep in this population may contribute to diminished attention and emotional instability (3). More critically, adolescent insufficient sleep, if left unaddressed, may persist into adulthood and demonstrate significant associations with obesity, metabolic syndrome, and mental health challenges (4).

Compared to other countries, sleep issues among Chinese children and adolescents are particularly pronounced, exhibiting dual characteristics of “short duration and poor quality.” Studies indicate that the prevalence of insufficient sleep in Chinese children and adolescents exceeds 60%, with an upward trend as age increases (5). In contrast, the average sleep duration among children and adolescents in Western countries is 1–2 h longer than their Chinese counterparts (6). The heightened likelihood of sleep problems in Chinese youth may be attributed to traditional cultural emphasis on academic achievement, intensive academic pressure from high-stakes examination systems (e.g., Zhongkao and Gaokao), disrupted circadian rhythms exacerbated by parental educational expectations in family structures, along with reduced sleep opportunities caused by after-school tutoring and widespread digital device usage (7).

Previous studies have demonstrated that excessive screen-based sedentary behaviors (SSB) are a significant contributing factor to insufficient sleep in children and adolescents (7). SSB refers to activities performed while seated or reclining that involve screen viewing (e.g., television, computers, mobile devices), excluding active gaming requiring physical movement (8). Besides increased insufficient sleep, excessive SSB are linked to inadequate physical activity, establishing a “sedentary-metabolic dysregulation” vicious cycle (9), increase risks of anxiety (10), and depression (11). Furthermore, SSB are associated with multidimensional health risks, including obesity, cervical disorders, and social withdrawal (12, 13).

Rapid advancements in screen technology, including smart TVs, computers, tablets, smartphones, virtual reality (VR), and augmented reality (AR), accompanied by significantly increased penetration rates of mobile devices (14), which has contributed to rising daily SSB among children and adolescents (15). These SSBs are no longer limited to traditional passive behaviors such as television watching or general computer use; instead, they have evolved into novel, mobile internet-driven forms—including short-video watching (e.g., on Douyin/Kuaishou), online gaming (e.g., multiplayer online battle arena games), online shopping (e.g., on Taobao/JD), and social media use via WeChat. Unlike traditional SSBs, these emerging behaviors are characterized by interactivity, algorithm-driven personalization, high immersion, and real-time social connectivity, which may differ in their mechanisms of influencing health outcomes such as sleep. The diversification of screen technologies and content formats has increasingly complicated the classification and definition of SSB (16), generating debates on the diverse effects of SSB on children and adolescents' health outcomes (17).

International studies reveal significant heterogeneity in the association between SSB and insufficient sleep: From usage typology, recreational screen time demonstrates particularly adverse effects, where each additional hour of recreational device use on weekends elevates adolescents' risk of delayed sleep onset by 30% (18), while educational active engagement (including online learning) shows no significant impact on sleep quality (19). For instance, emerging studies suggest that moderate use of educational interactive screens (including online learning platforms) may enhance sleep quality through cognitive stimulation (20), whereas passive video viewing shows significant associations with delayed sleep onset (19). Regarding gender differences, male adolescents exhibit significantly lower sleep adequacy rates due to online gaming preferences compared to females (21), whereas females experience a 5.3% decline in sleep efficiency for each additional hour of nighttime social media use (20). Although academic consensus on the strength of associations between SSB and sleep remains debated, relative agreement exists regarding gender-specific moderating effects (16).

The most recent survey indicates that with rapid advancements in internet technology, Internet penetration among Chinese children and adolescents has reached 97.2%, with smartphone ownership rates as high as 87.5% (14). Their daily screen time has increased significantly, centering around online learning, online gaming, short-video watching, and WeChat usage (22). Given the increasing prevalence of screen use among children and adolescents, patterns of sedentary behavior have undergone corresponding transformations. However, to date, no researchers have investigated the relationship between these emerging, mobile-centric SSBs—distinct from traditional television viewing or general computer use—and sleep outcomes in this population. Furthermore, academic consensus remains controversial and inconsistent regarding the associations between different types of SSBs and insufficient sleep, particularly lacking evidence on how these associations vary across demographic subgroups (e.g., gender, urban-rural residence, educational level). Therefore, this study aims to examine the specific associations between major forms of emerging SSBs and insufficient sleep among Chinese children and adolescents through two national cross-sectional surveys, and to explore whether these associations differ by gender, urban-rural status, and educational level.

## 2 Materials and methods

### 2.1 Data source and sample composition

The data utilized in this study were derived from the China Family Panel Studies (CFPS), funded by Project 985 of Peking University and conducted by the Institute of Social Science Survey (ISSS) at Peking University. Specifically, CFPS participants were selected from 25 provincial regions in China, including five large-sample provinces (Shanghai, Liaoning, Henan, Gansu, Guangdong) and 20 smaller-sample provinces (e.g., Jiangsu, Zhejiang, Fujian, Jiangxi, Anhui, Shandong, Hubei, and Beijing). The combined population of these regions represents ~94.5% of China's total population. CFPS employs population-proportional, multi-stage, implicit stratified sampling methods to ensure geographical representativeness. Given its comprehensive coverage and scientifically rigorous sampling design, the CFPS sample is considered highly representative of China's national population (23, 24). The CFPS includes detailed surveys of individuals, households, and communities. Individual-level data covers demographics, socioeconomic status, physical health, and psychological wellbeing, collected mainly through computer-assisted personal interviews. The CFPS also performs comprehensive post-survey verifications such as telephone validations, audio checks, and statistical analyses to ensure data accuracy (23, 24). CFPS data collection occurs every 2 years. Seven surveys were conducted between 2010 and 2022. Ethical approval was granted by the Ethics Committee of Peking University, and informed consent was obtained from all participants.

This study utilized data on children and adolescents aged between 10 and 18 years from the CFPS 2020 and 2022 waves (*n* = 6,051). To avoid biases due to missing data, participants with missing data on sleep (*n* = 1,545) or SSB (*n* = 1,018) were excluded. Additionally, participants missing data for key covariates—including urban/rural residence (*n* = 39), educational level (*n* = 137), exercise frequency (*n* = 1), academic pressure (*n* = 1), and interpersonal relationships (*n* = 1)—were also excluded. Consequently, the final analytical sample comprised 3,309 children and adolescents (female = 1,526; mean age = 14.12 ± 2.55 years).

### 2.2 Assessment of sleep

Sleep status was measured through a single questionnaire item assessing individuals' subjective evaluation of their sleep duration. The question was phrased: “Excluding nap time, how many hours do you usually sleep each day on average?” Respondents were instructed to report their average daily sleep duration numerically within the range of 0.1–24.0 h/day. Following recommendations from the American Academy of Sleep Medicine, which advises 8–12 h of sleep per 24 h for children and adolescents (25). This study operationalized insufficient sleep as < 8 h/day. Accordingly, participants reporting sleep durations of < 8 h/day were classified as having insufficient sleep, while those with ≥8 h/day were categorized as having sufficient sleep.

### 2.3 Assessment of screen-based sedentary behaviors

The frequency of five types of SSB—online gaming, online shopping, short-video watching, online learning, and WeChat usage—were assessed using CFPS items. Note that the CFPS questionnaire employs different recall periods for different SSBs, a design feature of the original dataset aimed at balancing recall accuracy (e.g., shorter periods for frequently occurring behaviors like online gaming, longer periods for less frequent or sustained behaviors like WeChat usage). For online gaming, online shopping, short-video watching, and online learning, respondents first answered questions such as: “In the past week, have you played online games?” Participants answering “yes” subsequently answered, “Did you play online games every day last week?” For WeChat usage, participants first answered, “In the past year, have you used WeChat?” If “yes,” they answered, “How frequently did you post updates about your work or life in your Moments (similar to Facebook or Instagram) in the past year?” In the current study, responses were coded as follows: a value of “1” indicated no participation in the specific SSB; “2” indicated occasional (non-daily) participation; and “3” indicated daily participation. In this study, responses regarding WeChat usage were coded to reflect different levels of platform engagement: a value of “1” indicated no use of WeChat in the past year; “2” indicated WeChat use without posting Moments updates (representing basic communication-only use); and “3” indicated WeChat use with posting of Moments updates (representing more active social presentation behavior).

### 2.4 Covariates

Based on previous literature and available CFPS data (26–30), the following covariates were included:

- Demographic covariates: gender, age, educational level, urban/rural residence.
- Lifestyle covariate: exercise frequency.
- Psychosocial covariates: academic pressure and interpersonal relationships.
- Health-related covariate: self-assessed health status.

Detailed variable definitions are provided in Table 1.

**Table 1:** The definitions of the key variables.

**Categories**	**Variables**	**Definition**
Dependent	Sleep	Sleep status was assessed using a single questionnaire item. A sleep duration < 8 h per day was defined as insufficient sleep ( coded as 1), while ≥8 h was classified as sufficient sleep (coded as 0).
Independent	Game	1 = Did not play online games in the past week; 2 = Played online games but not daily; 3 = Played online games daily.
	Shopping	1 = Did not shop online in the past week; 2 = Shopped online but not daily; 3 = Shopped online daily.
	Short video watching	1 = Did not watch short videos in the past week; 2 = Watched short videos but not daily; 3 = Watched short videos daily.
	Learning	1 = Did not engage in online learning in the past week; 2 = Engaged in online learning but not daily; 3 = Engaged in online learning daily.
	Wechat usage	1 = Did not use WeChat in the past year; 2 = Used WeChat but did not post Moments updates; 3 = Used WeChat and posted Moments updates.
**Controlled**		
Demographic	Age	Continuous variable, range: 10–18 years.
	Gender	Male = 1, Female = 0.
	Educational level	Elementary school = 3, Junior high school = 4, High school = 5.
	Urban/rural residence	Urban = 1, Rural = 0.
Lifestyle	Exercise frequency	Exercised ≥5 times per week = 0, Exercised < 5 times per week = 1.
Psycho-emotional	Academic pressure	Continuous variable, range: 1–5; higher scores indicate greater academic pressure.
	Interpersonal relationship	Continuous variable, range: 0–10; higher scores indicate better interpersonal relationships.
Health	Self-assessed health status	Excellent = 1; Very good = 2; Good = 3; Fair = 4; Poor = 5.

### 2.5 Statistical analyses

Descriptive statistics were conducted for SSB and sleep. Differences in SSB and sleep across groups with different covariates were assessed using *t*-tests (continuous variables) or chi-squared tests (categorical variables). Generalized linear logistic regression models were used to analyze associations between SSB and sleep, using three hierarchical models:

- Model 1: controlled covariates only (gender, age, educational level, urban/rural residence, academic pressure, interpersonal relationships, and self-assessed health status).
- Model 2: additionally included SSB (online gaming, online shopping, short-video watching, online learning, WeChat usage).
- Model 3: further included exercise frequency.

All analyses were conducted using Python and the statsmodels package (31). Statistical significance was set at a two-tailed alpha level of 0.05.

## 3 Results

### 3.1 Participants characteristics

Table 2 summarizes the demographic characteristics of the 3,309 participants included in this study. Participants had a mean age of 14.12 (SD = 2.55) years, with males comprising 53.88% of the sample. Elementary, junior high, and high school students accounted for 34.69, 34.63, and 30.67% of the sample, respectively. Rural participants made up 45.69%. The average scores for academic pressure, interpersonal relationships, and self-assessed health status were 2.74 ± 1.06, 6.92 ± 1.94, and 1.95 ± 0.89, respectively.

**Table 2:** Characteristics of participants according to cluster of sleep.

**Variable**	**Total**	**Total no (%)**	**Sufficient sleep**		***p*-Value from Chi-2/*t*-test**
			**Satisfied no (%)**	**Dissatisfied no (%)**	
Age	Mean (SD)	14.12 (2.55)	13.63 (2.52)	15.44 (2.12)	0.000
AP	Mean (SD)	2.74 (1.06)	2.65 (1.07)	2.96 (1.01)	0.000
IR	Mean (SD)	6.92 (1.94)	6.93 (1.96)	6.87 (1.91)	0.444
SH	Mean (SD)	1.95 (0.89)	1.88 (0.88)	2.12 (0.91)	0.000
Gender	Female	1,526 (46.12%)	1,091 (45.31%)	435 (48.28%)	0.624
	Male	1,783 (53.88%)	1,317 (54.69%)	466 (51.72%)	
EL	Elementary	1,148 (34.69%)	1,032 (42.86%)	116 (12.87%)	0.070
	Junior high	1,146 (34.63%)	833 (34.59%)	313 (34.74%)	
	High	1,015 (30.67%)	543 (22.55%)	472 (52.39%)	
Urban/rural	Urban	1,797 (54.31%)	1,326 (55.07%)	471 (52.28%)	0.216
	Rural	1,512 (45.69%)	1,082 (44.93%)	430 (47.72%)	
Exercise	≥5 times per week	1,786 (53.97%)	1,317 (54.69%)	469 (52.05%)	0.066
	< 5 times per week	1,523 (46.03%)	1,091 (45.31%)	432 (47.95%)	
**Independent**					
Game	No	1,285 (38.83%)	918 (38.12%)	367 (40.73%)	0.156
	Occasional	1,359 (41.07%)	1,014 (42.11%)	345 (38.29%)	
	Daily	665 (20.10%)	476 (19.77%)	189 (20.98%)	
Shopping	No	2,156 (65.16%)	1,678 (69.68%)	478 (53.05%)	0.100
	Occasional	1,114 (33.67%)	707 (29.36%)	407 (45.17%)	
	Daily	39 (1.18%)	23 (0.96%)	16 (1.78%)	
Short video	No	493 (14.90%)	356 (14.78%)	137 (15.21%)	0.005
	Occasional	1,420 (42.91%)	1,053 (43.73%)	367 (40.73%)	
	Daily	1,396 (42.19%)	999 (41.49%)	397 (44.06%)	
Learning	No	1,875 (56.66%)	1,375 (57.10%)	500 (55.49%)	0.430
	Occasional	927 (28.01%)	679 (28.20%)	248 (27.52%)	
	Daily	507 (15.32%)	354 (14.70%)	153 (16.98%)	
Wechat	No	517 (15.62%)	439 (18.23%)	78 (8.66%)	0.082
	No posted	2,745 (82.96%)	1,939 (80.52%)	806 (89.46%)	
	Posted on moments	47 (1.42%)	30 (1.25%)	17 (1.89%)	
Total		3,309 (100%)	2,408 (72.77%)	901 (27.23%)	

AP, academic pressure; IR, interpersonal relationship; SH, self-assessed health status; EL, educational level.

Regarding SSB, 20.10% reported daily online gaming, 1.18% engaged in daily online shopping, 42.19% watched short videos daily, 15.32% participated in daily online learning, and 1.42% had posted updates on WeChat Moments in the past year.

### 3.2 Univariate analyses

Table 2 presents comparative results between the sufficient sleep group and the insufficient sleep group. Significant differences were identified for age (*p* < 0.001), academic pressure (*p* < 0.001), self-assessed health status (*p* < 0.001), online shopping (*p* < 0.05), and short-video watching (*p* < 0.01).

### 3.3 Association between screen-based sedentary behaviors and sleep

Table 3 shows the associations between different SSB and sleep across three hierarchical logistic regression models.

**Table 3:** Associations between screen-based sedentary behaviors and sleep.

**Variable**	**Category**	**Model 1**		**Model 2**		**Model 3**	
		**OR**	**95% CI**	**OR**	**95% CI**	**OR**	**95% CI**
Age	9	Ref.		Ref.		Ref.	
	Each year increase	1.10^*^	[1.01, 1.19]	1.10^*^	[1.01, 1.19]	1.10^*^	[1.01, 1.19]
AP	1	Ref.		Ref.		Ref.	
	Each level improvement	1.19^***^	[1.10, 1.30]	1.19^***^	[1.09, 1.29]	1.19^***^	[1.09, 1.29]
IR	0	Ref.		Ref.		Ref.	
	Each level improvement	1.01	[0.96, 1.05]	1.00	[0.96, 1.05]	1.00	[0.96, 1.05]
SH	Excellent	Ref.		Ref.		Ref.	
	Each level decline	1.19^***^	[1.10, 1.30]	1.21^***^	[1.10, 1.33]	1.21^***^	[1.10, 1.33]
Gender	Female	Ref.		Ref.		Ref.	
	Male	0.96	[0.81, 1.13]	1.07	[0.89, 1.29]	1.08	[0.90,1.29]
EL	Elementary	Ref.		Ref.		Ref.	
	Junior high	2.33^***^	[1.67, 3.25]	2.35^***^	[1.68, 3.28]	2.34^*^	[1.68, 3.27]
	High	4.11^***^	[2.49, 6.77]	4.08^***^	[2.47, 6.74]	4.07^***^	[2.46, 6.73]
Urban/rural	Rural	Ref.		Ref.		Ref.	
	Urban	1.19^*^	[1.01, 1.40]	1.17	[0.99, 1.38]	1.16	[0.99, 1.38]
**Independent**							
Game	No			Ref.		Ref.	
	Occasional			0.81^*^	[0.67, 0.99]	0.81^*^	[0.67, 0.99]
	Daily			0.82	[0.64, 1.06]	0.82	[0.64, 1.06]
Shopping	No			Ref.		Ref.	
	Occasional			1.24^*^	[1.03, 1.49]	1.24^*^	[1.03, 1.49]
	Daily			1.48	[0.74, 2.95]	1.48	[0.74, 2.95]
Short video	No			Ref.		Ref.	
	Occasional			0.89	[0.69, 1.15]	0.89	[0.69, 1.15]
	Daily			0.64^***^	[0.49, 0.84]	0.64^***^	[0.49, 0.83]
Learning	No			Ref.		Ref.	
	Occasional			0.94	[0.78, 1.14]	0.94	[0.78, 1.14]
	Daily			1.02	[0.81, 1.29]	1.02	[0.81, 1.29]
Wechat	No			Ref.		Ref.	
	No posted			1.21	[0.91, 1.61]	1.21	[0.91, 1.61]
	Posted on moments			1.31	[0.65, 2.65]	1.32	[0.66, 2.66]
Exercise	≥5 times per week					Ref.	
	< 5 times per week					1.05	[0.89, 1.23]
Constant		0.02^***^	[0.01, 0.04]	0.02^***^	[0.01, 0.05]	0.02^***^	[0.01, 0.05]
Pseudo *R*^2^		0.12		0.13		0.13	
Observation		3,309		3,309		3,309	

AP, academic pressure; IR, interpersonal relationship; SH, self-assessed health status; EL, educational level.

^*^p < 0.05, ^**^p < 0.01, ^***^p < 0.001.

In Model 2, daily short-video watching (*p* < 0.001), online shopping (*p* < 0.05), and online gaming (*p* < 0.05) emerged as significant factors influencing children and adolescents' sleep. Specifically, compared to those who never watched short videos, participants engaging in daily short-video watching exhibited a 36% reduction in the odds of insufficient sleep (OR = 0.64, 95% CI: 0.49–0.84, *p* < 0.001). Conversely, daily online shopping was associated with a 24% increase in insufficient sleep odds relative to non-users (OR = 1.24, 95% CI: 1.03–1.49, *p* < 0.05), while daily online gaming showed a 19% decreased odds of insufficient sleep compared to non-gaming peers (OR = 0.81, 95% CI: 0.67–0.99, *p* < 0.05). After further controlling for exercise frequency in Model 3, daily short-video watching (*p* < 0.001), online shopping (*p* < 0.05), and online gaming (*p* < 0.05) remained statistically significant predictors of the outcome (Table 3 for details).

Among covariates in Model 3, each one-level increase in academic pressure was associated with a 19% increase in odds of insufficient sleep (OR = 1.19, 95% CI: 1.09–1.29, *p* < 0.001). Each one-level decline in self-assessed health status was associated with a 21% increase in odds of insufficient sleep (OR = 1.21, 95% CI: 1.1–1.33, *p* < 0.001).

### 3.4 Subgroup analyses

Tables 4–6 present subgroup analyses examining associations between screen-based sedentary and sleep across gender, urban/rural residence, and educational level.

**Table 4:** Associations between screen-based sedentary behaviors and sleep: subsamples by gender.

**Variable**	**Category**	**Gender**	
		**Female**	**Male**
Age	9	Ref.	Ref.
	Each year increase	1.35 (1.27, 1.43)^***^	1.33 (1.26, 1.40)^***^
AP	1	Ref.	Ref.
	Each level improvement	1.15 (1.02, 1.31)^*^	1.23 (1.10, 1.37)^***^
IR	0	Ref.	Ref.
	Each level improvement	1.01 (0.95, 1.08)	1.01 (0.95, 1.07)
SH	Excellent	Ref.	Ref.
	Each level decline	1.29 (1.13, 1.49)^***^	1.15 (1.02, 1.31)^*^
**Independent**			
Game	No	Ref.	Ref.
	Occasional	0.82 (0.63, 1.07)	0.82 (0.61, 1.10)
	Daily	0.88 (0.58, 1.33)	0.82 (0.59, 1.14)
Shopping	No	Ref.	Ref.
	Occasional	1.26 (0.97, 1.63)	1.17 (0.90, 1.52)
	Daily	1.30 (0.53, 3.18)	2.17 (0.70, 6.76)
Short video	No	Ref.	Ref.
	Occasional	1.13 (0.77, 1.65)	0.73 (0.52, 1.03)
	Daily	0.70 (0.47, 1.03)	0.59 (0.42, 0.85)^**^
Learning	No	Ref.	Ref.
	Occasional	0.93 (0.71, 1.23)	0.97 (0.74, 1.26)
	Daily	0.93 (0.66, 1.30)	1.15 (0.84, 1.58)
Wechat	No	Ref.	Ref.
	No posted	1.32 (0.86, 2.04)	1.22 (0.84, 1.76)
	Posted on moments	1.25 (0.50, 3.08)	1.78 (0.57, 5.54)
Exercise	≥5 times per week	Ref.	Ref.
	< 5 times per week	1.08 (0.85, 1.37)	1.05 (0.84, 1.32)
Constant		0.0 (0.00, 0.01)^***^	0.0 (0.00, 0.01)^***^
Pseudo *R*^2^		0.131	0.111
Observation		1,526	1,783

AP, academic pressure; IR, interpersonal relationship; SH, self-assessed health status; EL, educational level.

^*^p < 0.05, ^**^p < 0.01, ^***^p < 0.001.

**Table 5:** Associations between screen-based sedentary behaviors and sleep: subsamples by urban/rural.

**Variable**	**Category**	**Urban-rural**	
		**Urban**	**Rural**
Age	9	Ref.	Ref.
	Each year increase	1.39 (1.31, 1.47)^***^	1.29 (1.22, 1.36)^***^
AP	1	Ref.	Ref.
	Each level improvement	1.11 (0.99, 1.25)	1.27 (1.14, 1.42)^***^
IR	0	Ref.	Ref.
	Each level improvement	1.02 (0.95, 1.09)	1.00 (0.95, 1.07)
SH	Excellent	Ref.	Ref.
	Each level decline	1.30 (1.13, 1.5)^***^	1.16 (1.03, 1.32)^*^
**Independent**			
Game	No	Ref.	Ref.
	Occasional	0.76 (0.58, 1.01)	0.91 (0.71, 1.17)
	Daily	0.80 (0.57, 1.12)	0.90 (0.65, 1.25)
Shopping	No	Ref.	Ref.
	Occasional	1.23 (0.94, 1.62)	1.20 (0.94, 1.54)
	Daily	3.05 (1.11, 8.37)^*^	0.67 (0.21, 2.09)
Short video	No	Ref.	Ref.
	Occasional	0.85 (0.59, 1.23)	0.89 (0.63, 1.27)
	Daily	0.68 (0.47, 1)	0.60 (0.41, 0.86)^**^
Learning	No	Ref.	Ref.
	Occasional	1.14 (0.86, 1.52)	0.83 (0.63, 1.07)
	Daily	1.14 (0.81, 1.61)	0.98 (0.72, 1.35)
Wechat	No	Ref.	Ref.
	No posted	1.08 (0.71, 1.61)	1.44 (0.99, 2.1)
	Posted on moments^a^	1.04 (0.43, 2.54)	
Exercise	≥5 times per week	Ref.	Ref.
	< 5 times per week	1.09 (0.85, 1.39)	1.02 (0.81, 1.27)
Constant		0.0 (0, 0)^***^	0.0 (0, 0.01)^***^
Pseudo *R*^2^		0.152	0.099
Observation		1,512	1,797

AP, academic pressure; IR, interpersonal relationship; SH, self-assessed health status; EL, educational level.

^*^p < 0.05, ^**^p < 0.01, ^***^p < 0.001.

^a^Due to the limited number of rural participants who updated their WeChat Moments, these participants were combined with those who used WeChat without updating Moments for analysis.

**Table 6:** Associations between screen-based sedentary behaviors and sleep: subsamples by educational level.

**Variable**	**Category**	**Educational level**		
		**Elementary**	**Junior high**	**High**
Age	9	Ref.	Ref.	Ref.
	Each year increase	1.33 (1.11, 1.58)^**^	1.07 (0.95, 1.21)	0.93 (0.75, 1.14)
AP	1	Ref.	Ref.	Ref.
	Each level improvement	1.32 (1.11, 1.57)^**^	1.08 (0.95, 1.23)	1.02 (0.84, 1.24)
IR	0	Ref.	Ref.	Ref.
	Each level improvement	0.99 (0.91, 1.09)	1.01 (0.94, 1.09)	0.90 (0.80, 1.01)
SH	Excellent	Ref.	Ref.	Ref.
	Each level decline	1.14 (0.92, 1.41)	1.23 (1.06, 1.43)^**^	1.63 (1.28, 2.07)^***^
**Independent**				
Game	No	Ref.	Ref.	Ref.
	Occasional	1.35 (0.86, 2.11)	0.70 (0.52, 0.95)^*^	0.79 (0.59, 1.07)
	Daily	1.74 (0.95, 3.21)	0.65 (0.45, 0.95)^*^	0.84 (0.60, 1.19)
Shopping	No	Ref.	Ref.	Ref.
	Occasional	1.97 (1.19, 3.25)^**^	1.35 (1.02, 1.80)^*^	0.98 (0.75, 1.27)
	Daily	15.74 (2.13, 116.19)^**^	0.94 (0.25, 3.62)	1.02 (0.42, 2.47)
Short video	No	Ref.	Ref.	Ref.
	Occasional	0.52 (0.31, 0.86)^*^	1.14 (0.74, 1.76)	0.98 (0.64, 1.52)
	Daily	0.61 (0.34, 1.08)	0.97 (0.62, 1.52)	0.50 (0.33, 0.77)^**^
Learning	No	Ref.	Ref.	Ref.
	Occasional	1.09 (0.69, 1.73)	0.77 (0.56, 1.04)	1.07 (0.79, 1.44)
	Daily	0.96 (0.52, 1.76)	1.04 (0.70, 1.53)	1.05 (0.74, 1.47)
Wechat	No	Ref.	Ref.	Ref.
	No posted	0.90 (0.57, 1.41)	1.50 (0.97, 2.31)	0.91 (0.39, 2.13)
	Posted on moments	0.00 (0, inf)	2.94 (0.89, 9.66)	1.06 (0.32, 3.48)
Exercise	≥5 times per week	Ref.	Ref.	Ref.
	< 5 times per week	1.05 (0.7, 1.58)	0.95 (0.73, 1.24)	1.15 (0.89, 1.49)
Constant		0.0 (0.00, 0.02)^***^	0.06 (0.01, 0.38)^**^	0.53 (0.04, 6.81)
Pseudo *R*^2^		0.045	0.031	0.048
Observation		1,148	1,146	1,015

AP, academic pressure; IR, interpersonal relationship; SH, self-assessed health status; EL, educational level.

^*^p < 0.05, ^**^p < 0.01, ^***^p < 0.001.

- Gender Subgroup Analysis (Table 4):

Male children and adolescents who watched short-videos had significantly reduced odds of insufficient sleep by 41% compared to non-users (OR = 0.59, 95% CI: 0.42–0.85). However, this protective association was not observed among females.

- Urban-Rural Subgroup Analysis (Table 5):

Rural children and adolescents who watched short-videos had reduced insufficient sleep odds by 40% compared to non-users (OR = 0.60, 95% CI: 0.41–0.86). However, no such associations were found among urban children and adolescents.

- Educational Level Subgroup Analysis (Table 6):

Online shopping was no longer identified as a significant risk factor for insufficient sleep among high school students. Similarly, online gaming demonstrated no statistically significant associations with insufficient sleep in either elementary or high school student populations. Additionally, short-video watching was no longer a significant factor influencing insufficient sleep among junior high school students. Health status showed no significant association with insufficient sleep in primary school students. Interpersonal relationships demonstrated no significant correlation with insufficient sleep among elementary, junior high, or high school children and adolescents. Academic pressure exhibited no statistically significant relationship with insufficient sleep in in both junior high and high school populations.

## 4 Discussion

This study explored associations between various SSB and insufficient sleep among Chinese children and adolescents. Using nationally representative cross-sectional data from the 2020 and 2022 CFPS surveys, our findings indicated no significant associations between insufficient sleep and online learning or WeChat usage among children and adolescents. However, online gaming, online shopping, and short-video watching demonstrated significant associations with insufficient sleep. Specifically, online gaming and short-video watching exhibited negative correlations with insufficient sleep. For example, suggesting that short-video watching may serve as a protective factor against insufficient sleep among rural and male children and adolescents, while online gaming may have a protective effect among junior high school children and adolescents. Conversely, online shopping showed a positive correlation, indicating it may be a risk factor for insufficient sleep, increasing its likelihood.

Online learning refers to an educational approach utilizing internet and digital technologies, offering students flexibility in learning time and space. Previous studies have suggested that online learning may negatively impact university students' sleep, such as through circadian rhythm disruptions and reduced sleep quality (32). However, the present study found no association between screen-based online learning and insufficient sleep among children and adolescents. A plausible explanation lies in parental mediation in screen time management during home-based online learning (e.g., setting device usage schedules), which may effectively mitigate excessive nighttime screen exposure (33). Additionally, children and adolescents are in critical developmental stages with relatively higher sleep demands and more stable biological clocks and sleep-wake rhythms (34). Under structured daily routines and appropriate guidance, the impact of online learning on their sleep may be limited. Consequently, our findings revealed no significant association between online learning and insufficient sleep in this population. Future research should account for individual differences among children and adolescents to comprehensively elucidate the relationship between online learning and insufficient sleep.

WeChat is China's leading multifunctional social media platform, boasting over a billion monthly active users. Unlike international platforms such as Facebook, Twitter, or Instagram, WeChat primarily facilitates communication with acquaintances, akin to mobile text messaging, and interactions are largely confined to close friends or family members (35). Previous studies on the relationship between social media use and insufficient sleep remain inconclusive, with current consensus suggesting this relationship depends on platform-specific content and usage duration. Our study found no significant association between WeChat usage and insufficient sleep among children and adolescents. A key explanation lies in the specific coding of WeChat usage in this study: “posting updates in WeChat Moments”—the core indicator of active engagement we measured—involves social presentation and self-presentation attributes, but the participation rate of children and adolescents in this behavior is low, and most use occurs outside pre-sleep hours, failing to trigger significant pre-sleep arousal. In contrast, the instant messaging function of WeChat (e.g., homework discussions), which is the main usage scenario for children and adolescents, was not separately distinguished in our coding; its impact on sleep requires further verification in future studies.

Furthermore, stringent parental screen time supervision in Chinese households and the widespread adoption of technical controls such as WeChat's “Adolescent Mode” likely contribute to this null association by effectively regulating usage duration and timing. Collectively, these factors substantiate the lack of association between WeChat usage and insufficient sleep in this population.

Online gaming is defined as interactive recreational activities conducted via the internet. Previous studies have demonstrated a positive association between online gaming and insufficient sleep, particularly among adolescents, where excessive engagement may lead to reduced sleep quality and duration (36). However, this study found that online gaming served as a protective factor against insufficient sleep among junior high school students, while no statistically significant associations were observed in elementary or high school populations. This discrepancy may stem from the following mechanisms: Junior high school students, in early adolescence, exhibit significantly higher neural sensitivity to immediate gaming rewards than high school students (37). Neurodevelopmental studies have shown that the reward system (e.g., dopamine pathways) in early adolescence is more responsive to feedback and incentives, and moderate online gaming stimulation can regulate emotional states by fulfilling needs for autonomy and competence (self-determination theory), thereby contributing to improved sleep quality. Specifically, functional magnetic resonance imaging (fMRI) research has revealed that successful events in online games activate dopamine-related brain regions (e.g., caudate nucleus, orbitofrontal cortex), and this activation is associated with the regulation of reward prediction errors—suggesting moderate online gaming stimulation improves sleep quality through dopamine-mediated emotional regulation (38). Additionally, dopamine neurons encode not only expected reward value but also risk levels; the immediate feedback and variable rewards in online games enhance emotional regulation by modulating dopamine release, further supporting the protective effect of moderate online gaming on junior high school students' sleep (39). Moderate gaming stimulation may regulate emotional states, thereby contributing to improved sleep quality. Collaborative interactions within games can alleviate real-world social anxiety through in-game social engagement (40), potentially facilitating sleep. Due to younger age, elementary school students' online gaming behaviors are typically subject to strict parental supervision, consequently demonstrating no significant impact on insufficient sleep. Facing college entrance examination pressures, high school students often use online gaming as a strategic stress management tool rather than a risk factor for insufficient sleep (37). Therefore, we recommend that future interventions targeting online gaming behaviors prioritize elementary or high school student populations rather than junior high school students.

Online shopping is defined as the behavior of acquiring product information, making transactional decisions, and completing payments through internet platforms. While previous research has suggested a link between online shopping and insufficient sleep, particularly among adolescents, empirical evidence supporting this association remains limited. Our findings indicate that this association exists solely among elementary and middle school students, whereas online shopping does not constitute a risk factor for insufficient sleep in high school students. This discrepancy can be further explored from cognitive and developmental perspectives, with the neurodevelopmental characteristics of younger adolescents (elementary and middle school students) being a key explanatory factor—these students are more susceptible to instant gratification due to their immature neural inhibitory capacity—and cognitive maturation differences serving as another key explanatory factor. Neurodynamic studies have demonstrated that the brain's capacity to process immediate rewards gradually strengthens with age. Younger adolescents exhibit weaker neural inhibition toward stimuli such as “time-limited discounts” and “social sharing,” making them more prone to impulsive late-night purchasing behaviors that reduce sleep duration (41). This phenomenon likely stems from their underdeveloped prefrontal cortex, which heightens sensitivity to immediate rewards while compromising inhibitory control. Additionally, the maturation of executive functions in high school students aged 16–18, reaching near-adult levels (42), serves as another explanatory factor. Enhanced executive functioning enables better self-regulation and goal management, allowing high school students to incorporate recreational activities (including online shopping) into structured schedules while maintaining a balance between leisure and rest. This cognitive development facilitates more effective behavioral and temporal self-management, thereby mitigating potential sleep disruptions from online shopping. Consequently, we propose that future interventions targeting online shopping behaviors should prioritize elementary and middle school populations rather than high school students.

Short videos, typically lasting seconds to a few minutes, have become extremely popular due to their concise format, personalized recommendations, and influencer engagement, surpassing platforms like Facebook and Instagram (43, 44). Previous studies have linked short-video watching to insufficient sleep, especially among adolescents (45). However, our research indicates that short-video watching acts as a protective factor against insufficient sleep for elementary and high school students but not for junior high school students. This may be because Chinese parents use short videos as a “digital pacifier” for young children (46), and elementary school students, with limited energy, may find the audiovisual stimulation relaxing before sleep. For high school students, restricted access in dormitories may turn moderate viewing into a stress-relief mechanism, enhancing sleep quality (47). Specifically, an EEG study found that short video watching is associated with reduced prefrontal theta wave power (a marker of executive control load); its short-term, high-frequency stimulation diverts attention from stressors, achieving psychological relaxation and indirectly improving sleep quality (48). In contrast, junior high school students, who often use proactive strategies like social interaction and active activities to cope with stress, do not benefit from short-video watching in terms of sleep (49). Additionally, short video consumption is a protective factor against insufficient sleep for male and rural youth but not for female and urban populations. This may be due to male adolescents' engagement in highly interactive digital media, which provides positive social experiences and stress relief (50)—consistent with studies showing social media use buffers acute stress via social support or distraction effects (51). For rural youth, especially left-behind children, short videos provide emotional support and an enhanced sense of belonging (52), reducing loneliness and improving sleep quality (53), rural areas have relatively scarce entertainment resources, so short videos (as an easily accessible leisure option) further strengthen this protective association. Therefore, future interventions targeting short video use should prioritize junior high school students and female and urban children and adolescents.

To the best of our knowledge, this study represents the first investigation into associations between various contemporary SSB and sleep among Chinese children and adolescents. Key strengths include using nationally representative data and performing subgroup analyses across gender, urban-rural residence, and educational level.

Despite its contributions, this study has several limitations. First, as noted in detail earlier, the reliance on single-item, self-reported measures for sleep, SSB, and key covariates introduces potential for measurement error and bias, constraining the reliability and depth of our findings. Future studies should incorporate validated multi-item scales, objective sleep assessments (e.g., actigraphy), and objective or device-based measures of screen time. Second, the cross-sectional design precludes any causal inference. Longitudinal studies are necessary to elucidate the temporal ordering and potential bidirectional relationships between SSB and sleep. Third, the CFPS data did not provide precise information on the daily duration of screen time for each behavior, preventing dose-response analyses which could offer deeper insights. Future research should strive to collect detailed time-use data. Fourth, the landscape of digital technology evolves rapidly. The SSB types examined here (e.g., specific platforms) might change in relevance, and new forms (e.g., immersive VR/AR experiences, new social media platforms) will emerge, potentially influencing sleep through novel mechanisms. Future research needs to remain agile and adapt to these changes. Finally, while we examined several important subgroups, other potential effect modifiers (e.g., socioeconomic status, parenting styles) warrant investigation.

## 5 Conclusions

This study identified short-video watching, online shopping, and online gaming as key factors associated with sleep outcomes in Chinese children and adolescents, with notable variations across subgroups. Short-video watching exhibited a protective association against insufficient sleep among rural and male children and adolescents. Online gaming also showed a protective association, specifically among junior high school students. In contrast, online shopping emerged as a risk factor for insufficient sleep, particularly among elementary and junior high school students. Other SSB, such as the level of engagement with WeChat usage and online learning, showed no significant associations with sleep. These findings highlight the complex and nuanced relationship between contemporary SSB and sleep, underscoring the need for targeted, population-specific interventions rather than one-size-fits-all approaches. Future policies and research should focus on monitoring evolving SSB patterns, investigating the underlying mechanisms for these associations (especially the unexpected protective ones), and developing evidence-based interventions that consider the specific type of screen activity and the demographic context of the user.

## Data Availability

The original contributions presented in the study are included in the article/supplementary material, further inquiries can be directed to the corresponding author.
